# Bio-impedance method to monitor colon motility response to direct distal colon stimulation in anesthetized pigs

**DOI:** 10.1038/s41598-022-17549-6

**Published:** 2022-08-12

**Authors:** Yushan Wang, Po-Min Wang, Muriel Larauche, Million Mulugeta, Wentai Liu

**Affiliations:** 1 Department of Bioengineering, University of California, Los Angeles, Los Angeles, CA USA; 2 Vatche and Tamar Manoukian Division of Digestive Diseases, Department of Medicine, David Geffen School of Medicine, CURE: Digestive Diseases Research Core Center (DDRCC), Center for Neurobiology of Stress and Resilience (CNSR), University of California, Los Angeles, Los Angeles, CA USA; 3 VA Greater Los Angeles Healthcare System, Los Angeles, CA USA; 4 Department of Electrical and Computer Engineering, University of California, Los Angeles, Los Angeles, CA USA; 5 California NanoSystems Institute, University of California, Los Angeles, Los Angeles, CA USA; 6 Brain Research Institute, University of California, Los Angeles, Los Angeles, CA USA

**Keywords:** Enteric nervous system, Colon, Biomedical engineering

## Abstract

Electrical stimulation has been demonstrated as an alternative approach to alleviate intractable colonic motor disorders, whose effectiveness can be evaluated through colonic motility assessment. Various methods have been proposed to monitor the colonic motility and while each has contributed towards better understanding of colon motility, a significant limitation has been the spatial and temporal low-resolution colon motility data acquisition and analysis. This paper presents the study of employing bio-impedance characterization to monitor colonic motor activity. Direct distal colon stimulation was undertaken in anesthetized pigs to validate the bio-impedance scheme simultaneous with luminal manometry monitoring. The results indicated that the significant decreases of bio-impedance corresponded to strong colonic contraction in response to the electrical stimulation in the distal colon. The magnitude/power of the dominant frequencies of phasic colonic contractions identified at baseline (in the range 2–3 cycles per minute (cpm)) were increased after the stimulation. In addition, positive correlations have been found between bio-impedance and manometry. The proposed bio-impedance-based method can be a viable candidate for monitoring colonic motor pattern with high spatial and temporal resolution. The presented technique can be integrated into a closed-loop therapeutic device in order to optimize its stimulation protocol in real-time.

## Introduction

The colon is a vital part of the digestive system that performs essential functions including absorbing water, electrolytes, and vitamins; fermentation; and forming and propelling feces^1–3^. A wide range of diseases, such as chronic constipation, diarrhea, multiple sclerosis, spinal cord injuries, brain trauma, and Hirschsprung’s disease, lead to colonic malfunction^4–10^. Patients with colonic dysfunction suffer adverse effects in the quality of life both physically and emotionally^8,11,12^.

Electrical stimulation is a viable therapy for colonic motor disorders, particularly for patients who are refractory to traditional pharmaceutical treatments. As interest grows, a number of studies on colonic electrical stimulation have been conducted, which showed promising outcomes under different conditions^13–23^. In most of the current studies, the effectiveness of the technique was assessed mainly via colonic motility evaluation. This conventional way to assess the colonic motility uses a force/pressure transducer. However, there are limitations for a manometry approach, especially in colonic-related studies^24,25^. The insertion of the probes may cause uncomfortable sensations. Motion artifact may also interfere with the signals. Also, careful interpretation of the manometry data is required when contractions cause undetectable or very low luminal pressure changes. New techniques have been investigated to overcome these obstacles. For example, a proof-of-concept flexible piezoelectric device has been proposed for gastrointestinal (GI) motility sensing^26^, yet it still faces the complication due to motion artifact. Impedance measurement is another strategy to monitor GI motility. One of the bio-impedance techniques, placing electrodes on the abdomen and back, are based on the characteristics that impedance variation mainly depends on the changes in gastric volume due to food filling^27–31^. By measuring the bio-impedance in the gastric region, gastric motility and gastric emptying can be identified. However, it is more similar to general GI motility monitoring that consists of information from the whole GI sensitive region. Considering the complexity of colonic motility, it is difficult for this method to provide region-specific colonic motility types and patterns in detail. Another bio-impedance method is based on the tissue impedance variation due to the tissue deformation^32,33^, where a circuit system calculates the tissue impedance by applying a carefully filtered pulse width modulated voltage (at 50 kHz) to the tissue. The filtered signal mainly preserved 50 kHz component with a maximum peak-to-peak amplitude of 200 mV in order to prevent the undesired effects in the tissue such as contractility. The impedance is then deduced based on the current response to the filter signal. This method involves a tedious computation. Consequently, we proposed a novel method to monitor the colonic motility in porcine model by measuring colonic bio-impedance. It is an impedance measurement and analysis method using Randles Cell Model that has been validated in our prior studies on gastric motility measurements^34–37^. Different from the state-of-the-art bio-impedance methods, it is a hard-ware efficient method. It is shown that bio-impedance decreased significantly with strong colonic contraction during the stimulation in the distal colon, which is consistent with the earlier findings of the Mintchev group in^32,33^. Furthermore, the dominant frequencies (with most power/magnitude) of bio-impedance were identified and found to be shifted after the stimulation. The experimental results furthermore indicated the correlations between the proposed bio-impedance and the manometry approaches while dealing with the data reported in^23^, which demonstrates its potential application in colonic motility monitoring.

## Materials and methods

### Animals preparation and surgery procedure

In this study, male castrated mini-Yucatan pigs approximately seven months old and between 25 and 36 kg (S&S Farms, Ramona, CA) were used. All procedures were in accordance with the NIH Guide for the Care and Use of Laboratory Animals (8th edition), which were reviewed and approved by the UCLA Animal Research Committee (Institutional Animal Care and Use Committee) under protocol # 2018-074-01. All efforts were made to minimize any suffering and the number of animals used.

During the surgery, all pigs, in supine position, were intubated and given general anesthesia with 1–3% inhaled isoflurane. A midline abdominal incision was made. The proximal/ascending, transverse, and distal/descending colonic regions were identified and externalized. Each region had two customized-designed and fabricated planar electrode arrays^38^ placed on top of the serosal surface of the colon for bio-impedance recording and/or stimulation, as described in previous studies^37^. Flexible solid-state-manometry probes (Mikro-Cath™ diagnostic pressure catheter, ref 825–0101, Millar, Houston, TX) were inserted into the colon through a small incision and maintained in position using a loophole silk ligature for intracolonic pressure recording. Pigs were euthanized at the end of the experiment with an intravenous injection of pentobarbital (100 mg/kg, cat # 009444; Covetrus).

### Experiment protocol

**Figure 1:**
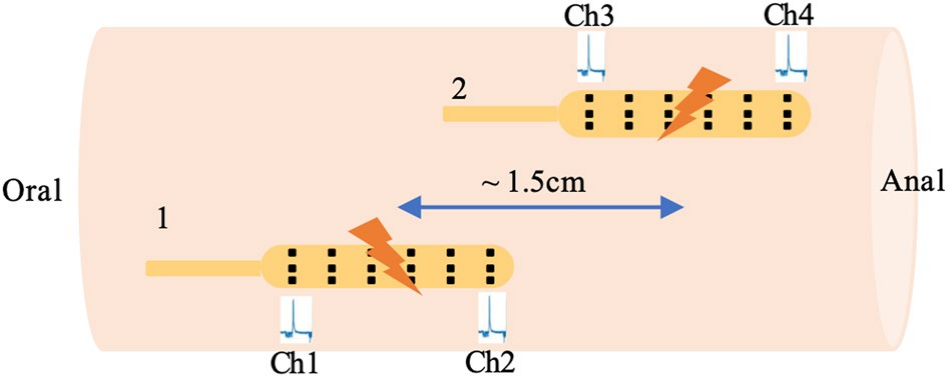
Experiment protocol. Direct distal colon stimulation: 5 cycles of alternating stimulation of electrodes #1 and #2 (total of 10 consecutive cycles) at 10 Hz, 2 ms, 15 mA, 30 s ON, 60 s OFF. Electrodes #1 and #2 were also used for recording, each containing four recording sites Ch1, Ch2, Ch3, and Ch4. The distance between the first two channels (Ch1–Ch2) and last two channels (Ch3–Ch4) is 1.5 cm, while Ch2 and Ch3 are separated by a distance of 3 mm.

The experiment consisted of a 30-min baseline, 15-min direct distal colon stimulation, and 30-min post-stimulation periods. A customized stimulation system^36,39^ was used for the stimulation. Two planar electrode arrays were placed 1.5 cm apart to stimulate alternatively (Fig. 1). The stimulation site was located in the center of each electrode. Based on the prior knowledge from trial studies under the same conditions^36,37^, the stimulation parameters were chosen as follows: A burst stimulation protocol composed of 10 Hz pulse train, 2 ms, 15 mA, at 30 s ON and then followed by 60 s OFF at each cycle. There are 10 consecutive cycles at a duration of 15 min. More details are available through Protocols.io^40^ and prior reported study^23^.

### Bio-impedance measurement

**Figure 2:**
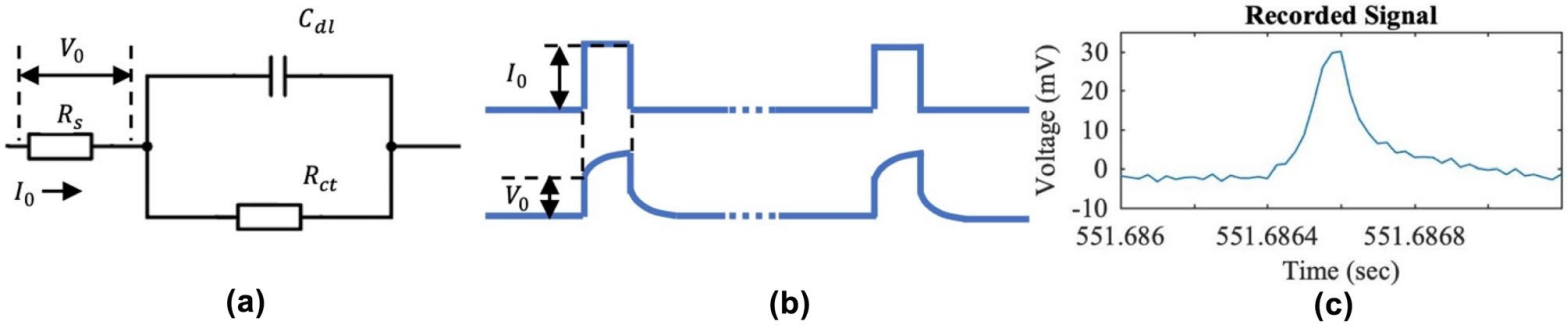
Bio-impedance mechanism. (**a**) Randles Cell Model. R_s_ reflects the colonic tissue impedance after stimulation. (**b**) Electrode overpotential resulted from a stimulus with small intensity. (**c**) Representative recorded signals.

The colonic wall bio-impedance changes reflect colonic motility since the tissue impedance changes with the smooth muscle deformation. With the basis of the Randles Cell electrode model (RC-model)^41–43^, a hardware-efficient bio-impedance method is proposed to monitor GI motility. The Randles cell model of an electrode, as shown in Fig. 2a, includes a tissue solution resistance ${\mathrm{R}}_{\mathrm{s}}$, a double layer capacitance ${\mathrm{C}}_{\mathrm{dl}}$, and a charge transfer resistance ${\mathrm{R}}_{\mathrm{CT}}$. Note that ${\mathrm{R}}_{\mathrm{s}}$ reflects the colonic muscle deformation measured in our case. By applying electrical stimuli of a series of narrow square pulses with low intensity into the colon, the response in terms of the RC-model can be obtained from the resulting electrode overpotential (Fig. 2b,c). Therefore, the electrode-tissue impedance can be concurrently extracted by ${V}_{0}/{I}_{0}$. The applied stimuli in our study were 200 Hz, 0.1 ms, 3μA, whose spectrum had a fundamental tone at 200 Hz and harmonics extended more than 10 kHz (1/0.1 ms). The RC-model responses were modulated by these high-frequency stimuli; as a result, the low-frequency (< < 200 Hz) motion artifacts can be filtered through a high-pass filter. It is important to note that the narrow and low intensity pulse must be well below the stimulation threshold charge of the colon according to the strength-duration curve. In our study, the current intensity is low enough (3μA in our case) so that it avoids activating the colon and directly interferes with the colonic motility. To avoid any confusion, “stimulation” in the rest of the paper refers to the burst stimulation protocol to activate direct distal colon. Without involving sophisticated computations, this method provides the bio-impedance information in a broad frequency spectrum. A similar technique for other applications has been validated in previous studies^35–37^.

Each colonic region (proximal, transverse, and distal colon) has two planar electrode arrays, each containing four recording sites along the longitudinal axis of the colon (Fig. 1). In each region from oral to anal, the distance between the first two channels (Ch1–Ch2) and last two channels (Ch3–Ch4) is 15 mm, while Ch2 and Ch3 are separated by a distance of 3 mm. The channels are accordingly denoted as P1–P4 for proximal colon, T1–T4 for transverse colon, and D1–D4 for distal colon. The electrodes were positioned to align with manometry probes, which will be described in the next subsection. The impedance data were recorded with a Plexon system (Plexon Inc., TX) at a sampling frequency of 40 kHz for the whole experiment including three separated intervals—namely baseline, stimulation, and post-stimulation intervals.

### Bio-impedance data analysis

The bio-impedance analysis is carried out at each interval. Both time domain analysis and dominant frequency components of the bio-impedance data were done by customized MATLAB codes. It should be noted that during “stimulation” interval, the bio-impedance analysis is intentionally done at the window of 60 s OFF time at each protocol cycle in order to avoid the complication of stimulation artifact.

Time domain analysis of the bio-impedance induced by the colonic phasic contractions were first performed. By quantifying the area under the curve of the bio-impedance change per minute, the colonic contractile-induced impedance changes (zAUC) were monitored and reported as bio-impedance motility index. Then the heatmap image is used to visualize the strength of average zAUC changes in percentage of baseline and their regional distribution across the colon.

The time domain signal was further divided into aforementioned separated intervals for frequency components analysis. The analysis is performed only for “baseline” and “post-stimulation” intervals. For the “stimulation interval”, considering the fact that the frequency resolution (Δf = 1/60 s = 1 cpm) corresponding to the short duration (60 s OFF time) is not fine enough for reliably analyzing the responses during “stimulation” interval, accordingly in this paper, our impedance analysis in frequency domain excludes the data extracted at the “stimulation” intervals. Discrete Fourier Transform (DFT) was applied to “baseline” and “post-stimulation” intervals to extract the frequency domain components, and the results were consequently compared. The conditional relationship between the frequency resolution and time window involved with DFT was met to ensure the accurate frequency analysis was obtained. The frequency with highest amplitude was identified as the dominant frequency components of the bio-impedance data, which reflects the frequency from which most contraction power originates.

### Assessment of intracolonic pressure

The intracolonic pressure signals were monitored for the whole experiment period, the same as the colonic wall bio-impedance measurement. Each colonic region has four manometric probes, each with a separation of 3 cm along the longitudinal axis. The proximal manometric probes were inserted about 10, 13, 16, and 19 cm below the ceco-colic junction, denoted as P10, P13, P16, and P19. The transverse manometric probes were inserted 10, 13, 16, and 19 cm after the end of proximal colon, denoted as T10, T13, T16 and T19. Distal probes were inserted through the anus with sensors at 10, 13, 16, and 19 cm proximal to the anal verge, denoted as D10, D13, D16, D19. The probes and the electrodes were manually aligned such that the electrodes were covered by the probes. The pressure signals were recorded at a sampling frequency of 100 Hz^23^. Motion artifacts induced by abdominal contractions and the high frequency noises were filtered by smoothing the raw recorded data with a time constant of 2 s and then removing DC components in the following data processing procedure^23,44^. The area under the curve of the filtered colonic phasic activity contraction was measured every minute (pAUC) and reported as manometry motility index. Correlation tests were then conducted between pAUC and zAUC.

### Statistical analysis

Considering the data properties and its complexity, the Generalized Estimating Equation (GEE) was used for statistical analysis^45–48^ on (1) changes of one-minute average impedance, expressed in percentage of baseline data, induced by stimulation; and (2) dominant frequency changes of impedance induced by stimulation. The statistical analyses were done for each channel separately. A P < 0.05 indicates a significant change.

### Animal experiment statement

This study follows the recommendations in the ARRIVE guideline. Yucatan minipigs, male castrated at 7 days of age (~ 7 months old, 25–36 kg, total of 7), were obtained from S&S Farms and group housed in pens (either bedding or grate floor, depending on housing availabilities—2 pigs/pen, 42 ft2) in an environmentally controlled room (lights on/off 6AM/6PM, 61–81°F) under specific pathogen-free conditions. All pigs received ad libitum access to diet (5p94 Prolab mini pig diet, PMI nutrition) and filtered tap water. All procedures were in accordance with the NIH Guide for the Care and Use of Laboratory Animals (8th edition), which were reviewed and approved by the UCLA Animal Research Committee (Institutional Animal Care and Use Committee) under protocol # 2018-074-01. During the surgery, all pigs, in supine position, were intubated and given general anesthesia with 1–3% inhaled isoflurane. Pigs were euthanized at the end of the experiment with an intravenous injection of pentobarbital (100 mg/kg, cat # 009444; Covetrus). All efforts were made to minimize any suffering and the number of animals used.

## Results

**Figure 3:**
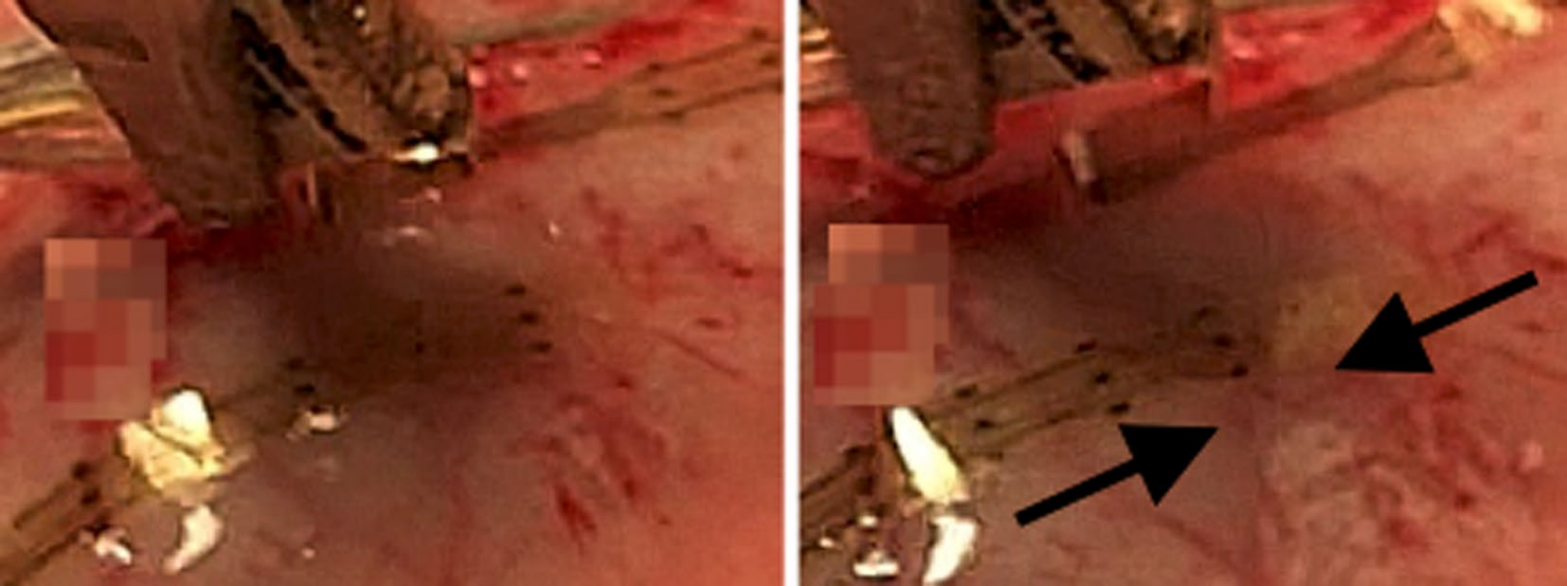
Longitudinal contraction of the distal colonic tissue in responses to direct *distal* colon stimulation. Left: Before stimulation. Right: During stimulation.

Direct colon stimulation did not cause hemodynamic changes. Direct distal colon stimulation resulted in an immediate local longitudinal contraction of the colonic tissue under the stimulation point (Fig. 3). The manometry results^23^ reported that it increased the distal colonic motility during and after the stimulation, while it caused less effects on proximal and transverse probes. Data associated with this study are available through the SPARC database^49^.

### Time course of bio-impedance changes

**Figure 4:**
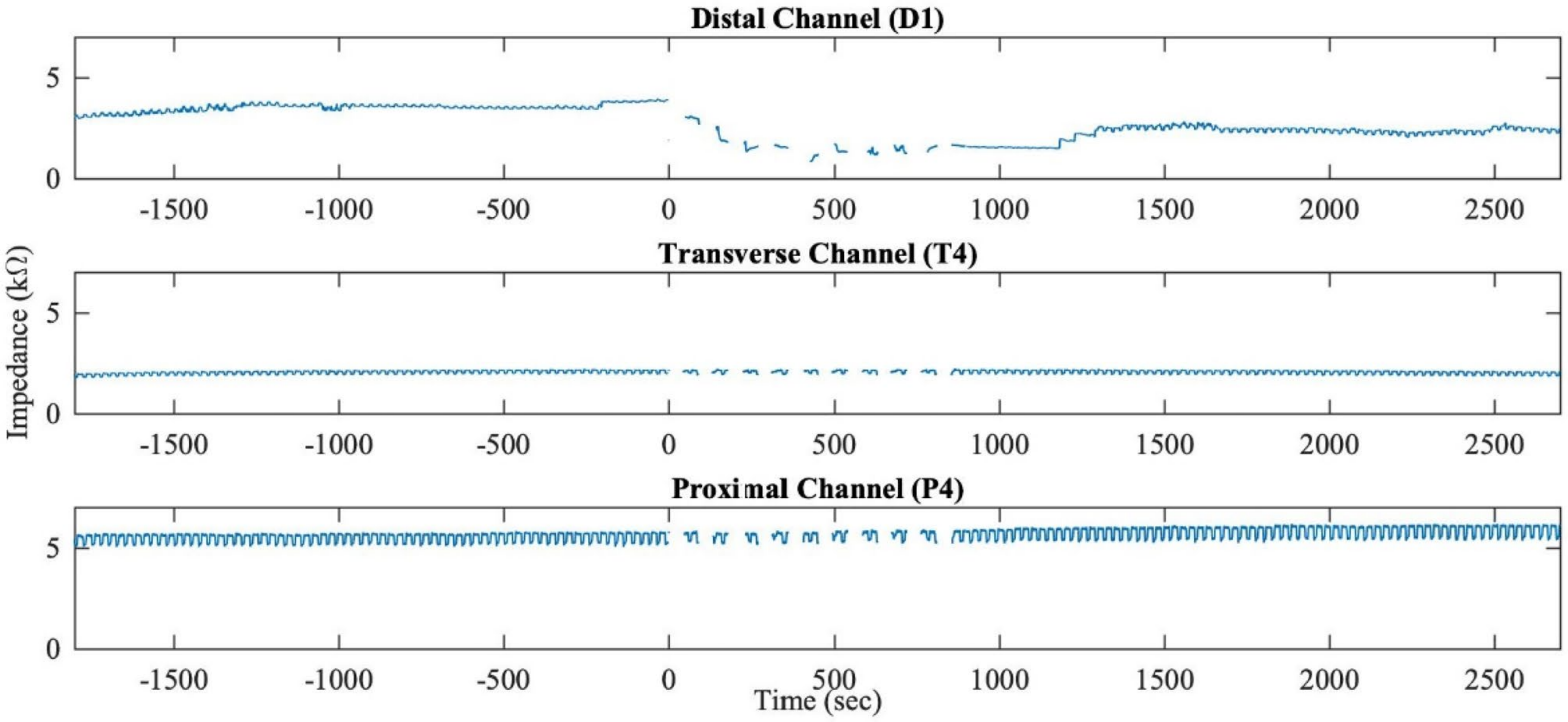
Example of different bio-impedance responses in time domain in proximal (P4), transverse (T4), and distal colon (D1). Stimulation starts at 0 s, and end at 900 s. Little or no DC changes in proximal and transverse colon while large impedance drop in distal colon during the stimulation, and gradually raise after the stimulation.

Figure 4 show representative bio-impedance responses of distal, transverse, and proximal channels in time domain. The bio-impedance generally exhibited a rhythmicity at around 2–3 cycles per minute (cpm) with additional DC drifts sometimes, of which the DC changes reflect the tonic contractile status. During the baseline periods from − 1800 to 0 s, the colonic bio-impedance remained stable for all three colonic regions. Corresponding with the activation of the electrodes on distal colon at 0 s, the local contraction occurred underneath the distal channels. The bio-impedance of the distal colonic tissue monitored by those channels decreased significantly. The large impedance drop was observed in most of the distal channels during the 15 min stimulation period. Table 1 shows the decreased bio-impedance (percentage of baseline) in all four distal channels, where the changes compared to baseline in both D2 and D3 channels reach statistical significances. After the stimulation, the bio-impedance gradually rise and become stable again in the majority of the distal channels. It is of interest that the bio-impedance of the D2 channel kept decreasing during the post-stimulation time. However, unlike the distal channels, the transverse and proximal channels showed variable increase and decrease during and after the stimulation, except for the significant increase in P4 during the stimulation and T2 after the stimulation. The change of DC component in the bio-impedance as a function of time reflected the colonic tissue motility. The colonic tissue contraction causes the decrease of bio-impedance, while the relaxation, on the contrary, causes the increase. This finding is consistent with previous studies^32,33,36,37^. An explanation for this phenomenon is based on resistance formula such that the contraction shortens the current path, and therefore decreases the impedance, while the tissue relaxation actually prolongs the pathway and increases the impedance.

**Table 1:** Colonic bio-impedance (in % baseline) during direct distal colon stimulation (15 min) and post-direct distal colon stimulation (30 min) in response to direct distal colon stimulation in anesthetized male Yucatan minipigs.

	n	Stimulation (in % baseline)	Post-stimulation (in % baseline)
P1	6	88.49 ± 19.69	93.04 ± 29.11
P2	6	95.18 ± 14.82	92.03 ± 31.06
P3	7	103.47 ± 13.54	103.58 ± 26.62
P4	5	112.62 ± 12.44**	108.10 ± 39.47
T1	6	91.07 ± 10.98	97.13 ± 23.33
T2	6	105.07 ± 9.68	115.41 ± 21.74
T3	4	111.21 ± 48.47	102.21 ± 65.33
T4	4	133.08 ± 35.75	127.96 ± 47.70
D1	6	83.69 ± 22.45	94.90 ± 23.50
D2	5	61.62 ± 23.63*	57.68 ± 24.98*
D3	4	59.17 ± 52.66*	86.44 ± 29.86**
D4	6	97.90 ± 19.91	106.57 ± 18.84

Data are mean ± SD of n as indicated for each channel. Significant changes (*P < 0.05 and **P < 0.01) were observed with respect to baseline impedance (100.00 ± 0.00%).

**Figure 5:**
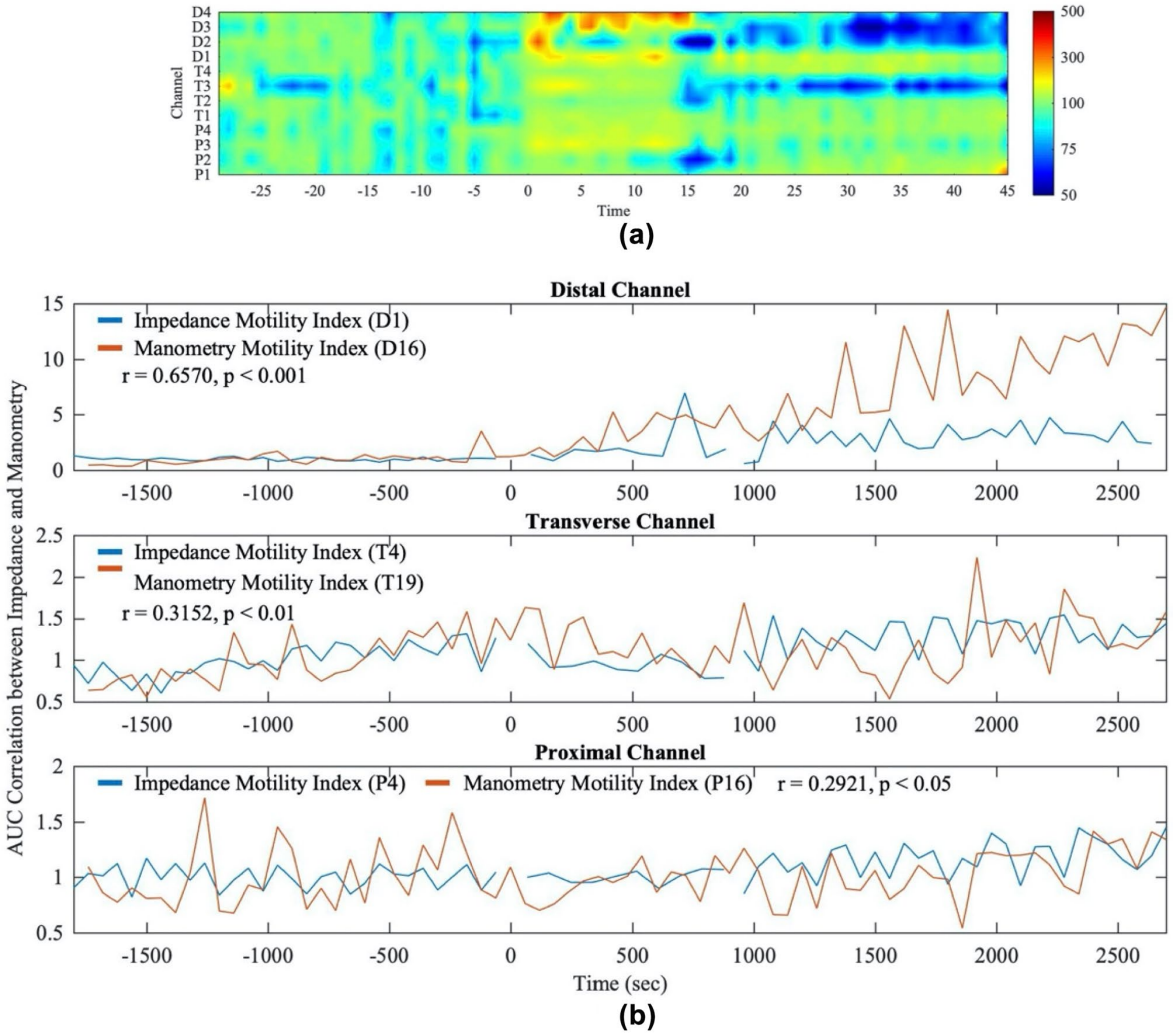
Bio-impedance motility and the correlation between the impedance motility index and manometry motility index. (**a**) Motility heatmap plot of bio-impedance. The color represents the percentage of the relative motility index change with respect to the baseline. The x-axis is time in minutes and y-axis is the channel index. (**b**) Representative results of AUC correlation between impedance channel and manometry probe in proximal (P4 and P16), transverse (T4 and T19), and distal colon (D1 and D16). Stimulation starts at 0 s, and end at 900 s.

As proposed in the previous method section, zAUC reveals the colonic phasic contraction-induced bio-impedance. The overall trend, shown in the bio-impedance heatmap (Fig. 5a), is that the stimulation of distal colon induced marked impedance changes of the distal colon, followed by fewer impedance changes for the next 30 min post-stimulation period. The impedance changes of proximal and transverse region were mostly less affected. To further test the correlation between the bio-impedance and manometry, the zAUC and pAUC of each channel/probe were compared. The results show that there exist high correlations between the manometry probes (P16, T19, D16) and impedance channels (P4, T4, D1) if their deployed positions are physically overlapped as the representative results shown in Fig. 5b, which indicates the bio-impedance method is correlated to the manometry information. However, we are aware of the existence of the uncorrelated channels and probes. It is important to note that manometry probes and bio-impedance recording channels employed different spatial resolutions. Consequently, there exist locations that manometry probes and bio-impedance recording channels are not aligned and overlapped in our experiments. As a result, the non-overlapping nature contributes to the uncorrelations. These different spatial locations can also explain the differences of the zAUC heatmap and the heatmap in the previous reported manometry^23^. The stimuli caused strong local activation of the recording region, which corresponded to the significant impedance changes. After the stimulation, the recording region gradually relaxed and became less active, while the contractile activity started a short propagation and caused colonic activation at the surrounding area. Therefore, the spatial resolutions with two different modalities may contribute to the differences between current bio-impedance and manometry data.

### Bio-impedance changes in frequency domain

**Figure 6:**
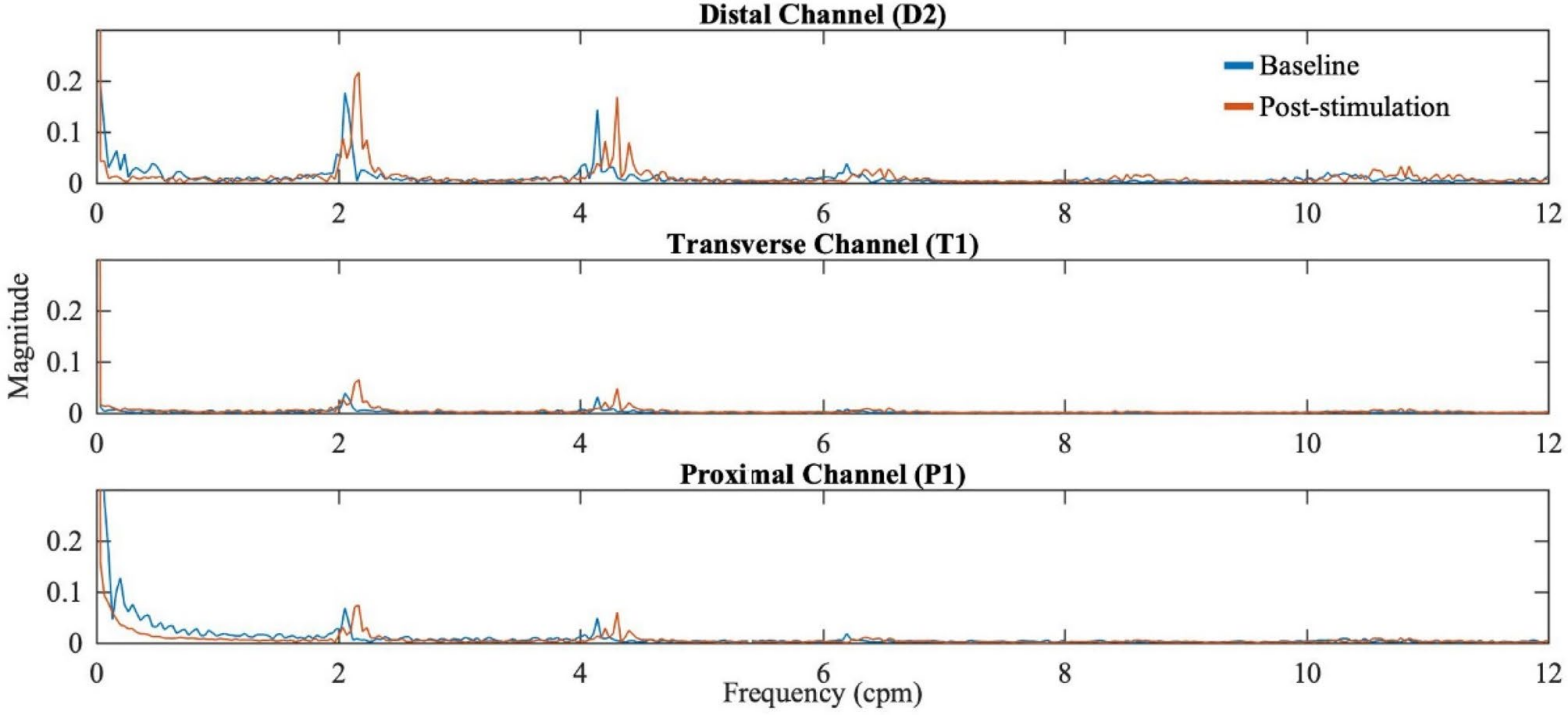
Example of different bio-impedance responses in frequency domain in proximal (P1), transverse (T1), and distal colon (D2). Peak frequencies are observed in the majority of the channels. The dominant frequency is in the range of 2–3 cpm. Dominant frequency (frequency with most power) shifts to the right significantly (P < 0.05) after the 15 min stimulation in most channels.

As described in our previously reported manometry results^23,50–53^, the frequency band of interest showing the phasic contraction lies in the range of 0–12 cpm. The ventilator/breathing frequency band is 13–17 cpm and the power of higher frequency band is negligible. Manometry data has a dominant frequency band of 1–6 cpm. However, the dominant frequency is difficult to be identified in the manometry data because of the lower resolution of manometric recordings and the dense peaks within 1–6 cpm. On the other hand, frequency spectrum analysis of the bio-impedance appears to be a better indicator for the phasic contraction. Clear dominant frequencies (frequencies from which most contraction power originates) of bio-impedance exist in the majority of the proximal, transverse, and distal channels during both baseline and post-stimulation period. Our results further narrowed down the range of dominant frequency to 2–3 cpm, which corresponded to the rhythmicity frequency that appeared in the aforementioned time course of bio-impedance changes. The high-resolution change of bio-impedance frequency further reveals the colonic motility profile. Figure 6 shows an example of the bio-impedance spectrum during baseline (blue) and post-stimulation (red) from one single experiment. The dominant peaks at around 2 cpm shifted to the right along the frequency axis after the stimulation, which is a sign of the activation effects of the colonic motility by stimulation as the higher frequency suggests stronger activities of the corresponding colonic region. In our study, the 15-min stimulation caused an average dominant frequency increase of 0.295 cpm with the highest value of 1.639 cpm. The shift (increase) in the dominant contraction frequency reaches a statistical significance in 7 out of 12 impedance channels (Table 2).

**Table 2:** Colonic bio-impedance dominant frequency during baseline and post-direct distal colon stimulation in anesthetized male Yucatan minipigs.

	Baseline (cpm)	Post-stimulation (cpm)
P1	2.2867 ± 0.2921	2.4232 ± 0.2967
P2	2.3054 ± 0.2624	2.325 ± 0.1705**
P3	2.3153 ± 0.2869	2.3472 ± 0.1675*
P4	2.2108 ± 0.2370	2.3206 ± 0.1885**
T1	2.4142 ± 0.2751	2.3758 ± 0.1788***
T2	2.3249 ± 0.2652	2.3272 ± 0.1891
T3	2.5158 ± 0.2353	2.403 ± 0.2871
T4	2.4108 ± 0.2717	2.5224 ± 0.4084
D1	2.31 ± 0.2765	2.3365 ± 0.1423**
D2	2.3151 ± 0.2722	2.3755 ± 0.1719*
D3	2.2462 ± 0.2069	2.4093 ± 0.2132***
D4	2.3071 ± 0.2582	2.3446 ± 0.1604

Data are mean ± SD of n as indicated for each channel. *P < 0.05, **P < 0.01 and ***P < 0.001 vs baseline.

## Discussion

In this study, we used a novel bio-impedance measuring approach to monitor colonic motility and found strong local effects of the direct distal colon stimulation in both time and frequency domain. We noted that the colonic bio-impedance drop of the distal channels correlated highly with the longitudinal contraction of the distal colon in response to the stimulation, which is intuitive as the longitudinal contraction, primarily the shrinking of the colon wall, shortens the current pathway and therefore reduces the bio-impedance. After the stimulation, the bio-impedance gradually increased, which matched with the colonic tissue recovery from the contraction status. The colonic bio-impedance of proximal and transverse channels presented divergent behaviors but were mainly inconspicuous compared to distal colon.

In the statistical analysis, we are aware of the limited pigs/channels with many consecutive measurements collected at different time separation in this study. Therefore, instead of the repeated measure analysis of variance (ANOVA), we applied GEE to make full use of the data and overcome the data collection problems mentioned above. The results showed that the bio-impedance changes reached statistical significances in D2 and D3 channels while most of the majority proximal and transverse channels did not. It is interesting to point out in our experiments, the statistical significances at each channel depends on the degree of colocalization between the positions of stimulation sites and recording channels. As shown in Fig. 1, stim site 1 was between D1 and D2, stim site 2 was between D3 and D4. Two stim sites were stimulated alternatively. When stim site 1 was on, the strongest effects are at D1 and D2, while D3 had stronger effects than D4 since D3 was closer to the stim site 1. This was true when stim site 2 was on. Therefore, D2 and D3 had more variations than D1 and D4 in general.

The heatmap shown in Fig. 5a derived from zAUC further highlighted the direct distal stimulation effects on the colonic phasic activities. Similar to the previously reported manometry findings^23^ by our group, bio-impedance method showed that the stimulation induced increased motility index in distal colon but varying fewer responses in proximal and transverse colon. However, there is a different interpretation for the distal channel reaction in post-stimulation duration. Manometry data showed strong activation of contraction while bio-impedance indicated inhibition. The key reason for the contrast is the positions of the manometry probes and impedance electrodes in both modalities. There is no consistent one to one position overlapping between the impedance electrode and manometry probe mainly because of mismatch in size and pitch as well as the difficulty involved in surgery. In fact, the spatial resolution (pitch) of the manometry probe is 3 cm, which is indeed a limitation in our study. The bio-impedance nodes had finer spatial resolution. Four bio-impedance channels in all the experiments were placed close to the stimulation electrodes, mainly monitoring the stimulation zone, while manometry probes covered larger areas away from the stimulation sites. The Bio-impedance provided more information about the colonic motility around the stimulation site and manometry provided more information of several centimeters away from the stimulation sites. Therefore, the two results are complementary instead of opposite. Combining the results of the two heatmaps, it is found that the stimulation site relaxed after the strong contraction and the stimulation might induce short propagation of the phasic contractions from the stimulation site to the nearby region after the stimulation. Moreover, consistencies were found between zAUC and pAUC in some channel-probe pairs (such as D1–D16 and T4–T19 shown in Fig. 5b) for each subject from the correlation studies. When bio-impedance and pressure sensors were placed close to each other, high correlations could be observed, while the correlation decreased when the separation distance increased because of the high spatial variations in colonic motility. Our results further support the previous studies that the bio-impedance decreases when the contraction happens^32,33,36,37^ and show the capability of bio-impedance approach to monitor the luminal pressure changes. Given that direct colon surface stimulation, using similar parameters as the current study, did not cause any histological damage^23^, the observed impedance change is unlikely to be confounded by tissue inflammatory response reported in ischemic small intestine^54^.

The bio-impedance responses in time domain indicate its potential to detect colonic contraction/expansion, and as a result, to monitor the colonic motility changes. This proposed technique has several advantages. For example, its higher temporal and spatial resolution can support more precise motility monitoring. The use of customized high density stretchable planar electrodes can cover specific area and provide more accurate spatial contraction information, which makes it a better way to capture and analyze the contraction propagation in specific directions. The impedance monitoring system can not only provide the colonic motility information, but also provide other information, such as whether the electrode overpotential is within its water window (the electric potential range which water is neither oxidized nor reduced at the electrode interface) and whether the electrode is well contacted to colonic tissue to track the stimulation safety and effectiveness^55–58^.

At baseline the dominant distal colon contraction frequency from which most contraction power comes from is in the range of 2–3 cpm. Following stimulation, the dominant frequency shifted to the higher baseline cpm range, suggesting that stimulation increases the magnitude/power of contractions higher than the baseline. Compared to the conventional manometry methods, the bio-impedance method can record the full frequency spectrum of the colonic contractions without losing the DC/low frequency components because of the noise and artifacts. While recording the evoked electrode overpotential at the interface of electrodes and colonic tissue, all frequency components of the tissue bio-impedance were modulated by the main tone at 200 Hz when the stimuli (200 Hz, 0.1 ms, 3 μA) is applied. Consequently, low frequency components are preserved at the sidebands and cleaner signals can be obtained via signal processing. Besides the dominant frequency in the range of 2–3 cpm, we also observed the spikes beyond that range, as ~ 4 cpm and ~ 6 cpm shown in Fig. 6. However, we suspect them to be the 2nd and 3rd harmonics of the dominant frequency (~ 2 cpm). Currently, the frequency analysis of the bio-impedance *during stimulation* (15 min) is not provided because of concerning the signal quality during this period; nevertheless, the analysis is possible in the duration of stimulation if proper selections of the stimulation parameters such as pulse width, frequency, and amplitude for bio-impedance method, as well as employing sophisticated techniques for stimulation artifact removal^59^.

Of note is that the colon is known to have different ICC-pace setting frequencies depending on the ICCs geographical location, ranging from 1 cpm to up to 30 cpm^60^ that lead to different contraction patterns. Neural activities get superimposed on slow waves to increase contraction amplitudes and modulate motility patterns. In pigs and the human colon, the dominant contraction frequency band from which most of the power (magnitude) of contraction comes from the 0–6 cpm band^23,52,53^. In the current data shown, of the prominent 0–12 cpm colon contractile events, electrical stimulation increases the power/magnitude of the dominant 2–3 cpm band.

The current study did not address the specific mechanism through which electrical stimulation caused the local (distal) and distant colon sites (proximal and transverse) contractile responses of the colon. However, direct gut tissue electrical stimulation is bound to activate smooth muscle cells, enteric nervous system, and extrinsic fibers within the tissue (primary afferent). Thus, the activation of colon smooth muscle cells, excitatory enteric neurons, and extrinsic parasympathetic fibers (vagal and lumbosacral) cause membrane depolarization and induction of action potentials that lead to colon contraction. On the other hand, activation of extrinsic sympathetic fibers in the colonic wall causes inhibition of motility and changes in intestinal blood flow^61–64^. The mechanism of sympathetic induced colon motility inhibition involves inhibition of myenteric excitatory neurons^65^. In addition, given that enteric glia cells are activated by sympathetic fibers through ATP^66^ and glia are known to regulate intestinal motility^67^, an indirect effect of sympathetic activation on motility can occur. Taken together, the net effect of direct colon electrical stimulation, using the stimulation parameters in the current study, is contraction at the stimulation sites as was shown in the current study through colon wall impedance monitoring and visual observation.

## Conclusion

This paper presents a novel bio-impedance method based on Randles Cell model to monitor the colonic responses to direct distal colon stimulation. It shows that the distal colon contraction regulates the tissue’s bio-impedance. The dominant (most power/magnitude) frequencies of the contractions have also been detected, which increased after the stimulation in most impedance recording channels. The post-stimulus increase in the magnitude of frequencies indicates an increase in colonic motility. Moreover, statistical analysis shows correlations between the colon motility monitoring modalities used bio-impedance and manometry. This is evidenced by the data when both the impedance electrodes and manometry probes are aligned in our study. In summary, the results of the study demonstrate the potential of bio-impedance method as a viable alternative of monitoring colonic motility.

## Data Availability

The datasets generated and/or analyzed during the current study are available in the SPARC database repository (https://sparc.science/datasets/34?type=dataset), 10.26275/up27-ibcr.
